# Speech‐in‐noise processing in Alzheimer's disease and primary progressive aphasia

**DOI:** 10.1002/alz70856_099262

**Published:** 2025-12-24

**Authors:** Sophie A Froud, Benjamin A Levett, Lucy B Core, Jessica Jiang, Charles R Marshall, Jason D Warren, Chris JD Hardy

**Affiliations:** ^1^ Dementia Research Centre, UCL Queen Square Institute of Neurology, London, London, United Kingdom; ^2^ Queen Mary University of London, London, Greater London, United Kingdom

## Abstract

**Background:**

In daily life, understanding spoken messages generally requires decoding of speech signals embedded in variably noisy acoustic backgrounds. This is a computationally demanding neural task that is likely to be vulnerable early in the course of neurodegenerative brain pathologies. However, how speech‐in‐noise processing is affected in different dementia syndromes, how it relates to peripheral hearing loss and the brain basis for impaired speech‐in‐noise perception remain unclear. Here we addressed these questions using a speech‐in‐noise test in a well‐defined cohort of patients with Alzheimer's disease (AD) and language‐led dementias (primary progressive aphasia (PPA) syndromes).

**Methods:**

We administered an in‐house Digit Triplets test in which participants listened binaurally to three digits presented in competition with speech‐weighted white noise and reported the digits heard on each trial. Signal‐to‐noise ratio (SNR) was adjusted from trial to trial based on task performance following an adaptive staircase protocol, and mean SNR for speech comprehension was generated for each individual. 48 patients (15 with AD, 13 with logopenic variant (lv)PPA, 12 with semantic variant (sv)PPA, eight with nonfluent/agrammatic variant (nfv)PPA) and 22 cognitively‐well, age‐matched older individuals participated. Peripheral hearing function as indexed on pure tone audiometry and general cognitive functions were assessed using standard test procedures. Clinical diagnosis of dementia syndromes was supported on volumetric brain MRI and voxel‐based morphometry (VBM) was used to identify structural neuroanatomical associations of speech‐in‐noise performance in the patient cohort.

**Results:**

The AD, lvPPA and nfvPPA groups had significantly higher mean SNR thresholds (performed significantly worse) than cognitively‐well older listeners on our speech‐in‐noise task, while the svPPA group did not significantly differ from healthy controls. SNR was at a significantly higher threshold in the lvPPA group than in all other syndromic groups. Auditory cortical correlates of speech‐in‐noise performance were identified on VBM in the patient cohort.

**Conclusion:**

AD and PPA syndromes are associated with separable profiles of speech‐in‐noise impairment, which appear to capture auditory brain dysfunction in these canonical dementia syndromes.

